# Reprogramming the gut–brain axis in neurodegeneration: comparative microbiome, metabolite, immunometabolic, polyphenol, probiotic, and epigenetic mechanisms in Alzheimer’s and Parkinson’s diseases

**DOI:** 10.3389/fnagi.2026.1865514

**Published:** 2026-10-23

**Authors:** Yu Jie Cheng, Mei Ping Tao, Kexin Qian, Wei Ye

**Affiliations:** 1 College of Post and Telecommunication of Wuhan Institute of Technology, College of Chemical and Materials Engineering, Wuhan, Hubei, China; 2 Department of Gastroenterology, Xiangshan Hospital of Traditional Chinese Medicine Medical and Health Group, Ningbo, Zhejiang, China; 3 Department of Gastroenterology, Hangzhou TCM Hospital, Hangzhou, China

**Keywords:** Alzheimer’s disease, curcumin, dietary bioactives, epigenetic regulation, gut microbiome, gut–brain axis, microbial metabolites, nanoparticles

## Abstract

The gut–brain axis has emerged as a central mechanistic framework linking dietary bioactives, microbial ecology, and neurodegenerative disease progression. Similar microbiome-related mechanisms have also been implicated in colorectal cancer, suggesting potential shared links between microbial dysbiosis, inflammation, and disease progression. This study synthesizes preclinical evidence examining how polyphenols, polyphenol-derived compounds, and advanced delivery systems, including nanoparticles and synbiotics, modulate Alzheimer’s disease (AD) and Parkinson’s disease (PD) through microbiome-dependent mechanisms. Evidence from transgenic mice, toxin-induced rodent models, and *Drosophila* systems was compared across microbiome alterations, microbial metabolites, signaling pathways, epigenetic regulation, and functional outcomes. Across AD and PD models, dietary bioactives consistently promoted restoration of gut microbial homeostasis, characterized by enrichment of beneficial taxa and reduction of dysbiosis-associated microorganisms. These changes were accompanied by alterations in microbial metabolites, particularly short-chain fatty acids (SCFAs), tryptophan-derived metabolites, and lipid-associated signaling molecules. These metabolites provide functional links between microbial ecology and CNS physiology and may regulate epigenetic processes, including histone acetylation, DNA methylation, chromatin remodeling, and non-coding RNA activity. Despite substantial upstream convergence, downstream effects showed disease-specific patterns. In AD models, microbiome modulation predominantly influenced metabolic, amyloidogenic, and neurotrophic pathways, including AKT/GSK3β, NRF2, NF-κB, and BDNF-associated signaling, contributing to reduced amyloid-β deposition, attenuated tau pathology, and improved cognitive outcomes. In PD models, greater emphasis was placed on inflammatory, oxidative, and mitochondrial pathways involving SIRT1/NRF2, TLR4/MyD88/NF-κB, inflammasome signaling, and neuronal survival mechanisms, resulting in reduced neuroinflammation and preservation of dopaminergic neurons and motor function. Epigenetic regulation represents an additional convergence layer through which microbial metabolites and polyphenols may influence inflammatory, metabolic, mitochondrial, and neuronal gene programs in both disorders. Nanoparticle-based delivery systems enhanced bioavailability, tissue targeting, and microbiome-related effects, whereas synbiotic strategies reinforced microbial and metabolic homeostasis. Collectively, these findings support a microbiome–metabolite–epigenome–brain framework in which dietary bioactives modulate neurodegeneration through interconnected microbial, metabolic, immune, redox, mitochondrial, and epigenetic pathways. This framework provides a mechanistic basis for developing disease-tailored and precision-oriented gut–brain interventions for AD and PD.

## 1 Introduction

Neurodegenerative disorders such as Alzheimer’s disease (AD) and Parkinson’s disease (PD) are now recognized as multisystem conditions shaped not only by central pathology but also by peripheral ecological and metabolic disturbances (Fan et al., 2026; Jin et al., 2026a; Phillips and Picard, 2024). Across diverse models, gut dysbiosis, impaired microbial metabolite production, and chronic low-grade inflammation reproducibly accompany and often precede hallmark neuropathology (Czaj et al., 2026; Jin et al., 2026b; Schoultz et al., 2025; Shen et al., 2025). These findings position the gut-brain axis as a mechanistic interface linking systemic homeostasis to neurodegenerative vulnerability. This network integrates microbiota composition, intestinal barrier integrity, microbial metabolites, immune signaling, and neuroendocrine pathways.

Preclinical evidence consistently identifies disease-associated microbial signatures. In PD models, including MPTP- and rotenone-induced mice, dysbiosis is characterized by an expansion of pro-inflammatory taxa and a reduction in SCFA-producing bacteria. These microbial alterations are associated with intestinal barrier dysfunction, microglial activation, and dopaminergic neuronal degeneration (Cai et al., 2023; Tao et al., 2023; Zhong et al., 2022). Similarly, AD models such as APP/PS1 and 5xFAD mice show pronounced ecological disruption linked to amyloid-β (Aβ) accumulation, oxidative stress, and cognitive decline (Lv et al., 2018; Su et al., 2022; Upadhyay et al., 2024). Although these disorders differ in their predominant neuropathology, characterized by Aβ and tau pathology in AD and α-synuclein accumulation with dopaminergic neuronal loss in PD, they exhibit strikingly similar microbial and metabolic disturbances (Guerrero-Torres et al., 2026; Jin et al., 2026b; Lehoczki et al., 2025).

Within this mechanistic landscape, dietary bioactive compounds, especially polyphenols, emerge as reproducible first-line modifiers of gut ecology and systemic inflammation (Zekrumah et al., 2023). In PD models, curcumin restores microbial balance, enhances SCFA levels, repairs intestinal barrier function, and preserves dopaminergic neurons across multiple toxin-based paradigms (Cai et al., 2023; Cao et al., 2024; Zhong et al., 2022). Resveratrol similarly steers microbiota profiles toward less inflammatory configurations in MPTP/P-induced PD, improving behavioral and neuroinflammatory outcomes (Tao et al., 2023). These findings indicate that polyphenols operate not only as direct antioxidants but as upstream ecological modulators with system-wide metabolic and immune consequences (Wang, 2026; Zafari et al., 2025; Zhang et al., 2022).

Comparable mechanistic patterns are observed in AD. Resveratrol-loaded nanoparticles reduce insulin resistance, remodel gut microbial communities, and attenuate Aβ and tau pathology via the AKT/GSK3β axis (Liu et al., 2025; Rey et al., 2019; Yang et al., 2023a,b). These data suggest that metabolic dysfunction, particularly impaired insulin signaling and lipid homeostasis, is deeply integrated with AD pathogenesis and can be modulated indirectly through microbiota-directed interventions (Yang et al., 2023a).

Advanced nanodelivery systems further amplify these effects. Brain-targeted resveratrol-chitosan nanoparticles enhance bioavailability, improve cognition, normalize microbial communities, and reduce neuroinflammation in AD models (Kamath et al., 2024; Rajkumar et al., 2026; Yang et al., 2023a). Here, delivery technology functions as a mechanistic amplifier, enabling polyphenols to more effectively reshape the ecological and metabolic layers of the gut–brain axis.

A broader range of polyphenols and traditional formulations reinforce this microbiota-centered model. Quercetin increases beneficial taxa and enhances brain-derived neurotrophic factor (BDNF) related neurotrophic signaling in vitamin D-deficient APP/PS1 mice (Lv et al., 2018). Anthocyanins from *Lycium ruthenicum* reduce neuroinflammation and motor deficits in PD through coordinated microbial and metabolomic remodeling (Cao et al., 2024). Triphala, a polyphenol-rich formulation, normalizes gut microbial communities and improves cognition in AD models (Upadhyay et al., 2024). Marine polyphenols from *Sargassum wightii* undergo microbial biotransformation into more potent antioxidant and acetylcholinesterase-inhibitory metabolites, highlighting the microbiota as a metabolic bioreactor that determines therapeutic efficacy (Gade and Kumar, 2025).

Microbiota-dependence studies provide direct mechanistic evidence. Synbiotic interventions in Drosophila AD models delay pathology through modulation of PPARγ signaling, oxidative stress, and immune activity (Westfall et al., 2019). Conversely, antibiotic depletion abolishes the neuroprotective effects of EGCG in PD, demonstrating that gut bacteria are essential mediators of phytochemical bioactivity (Xu et al., 2020). Such findings move the field beyond correlation and confirm causal microbiota contributions to neuroprotection.

Collectively, AD and PD share a systems-level pathological architecture defined by gut dysbiosis, impaired SCFA and metabolite production, metabolic instability, and chronic neuroinflammation (Figure 1). Whereas AD is dominated by Aβ/tau pathology and insulin resistance, and PD by α-synuclein aggregation and dopaminergic loss, both disorders exhibit upstream ecological and metabolic disruption that is amenable to intervention. Restoration of microbial balance consistently aligns with improvements in central pathology, immune regulation, and behavioral outcomes.

**FIGURE 1:**
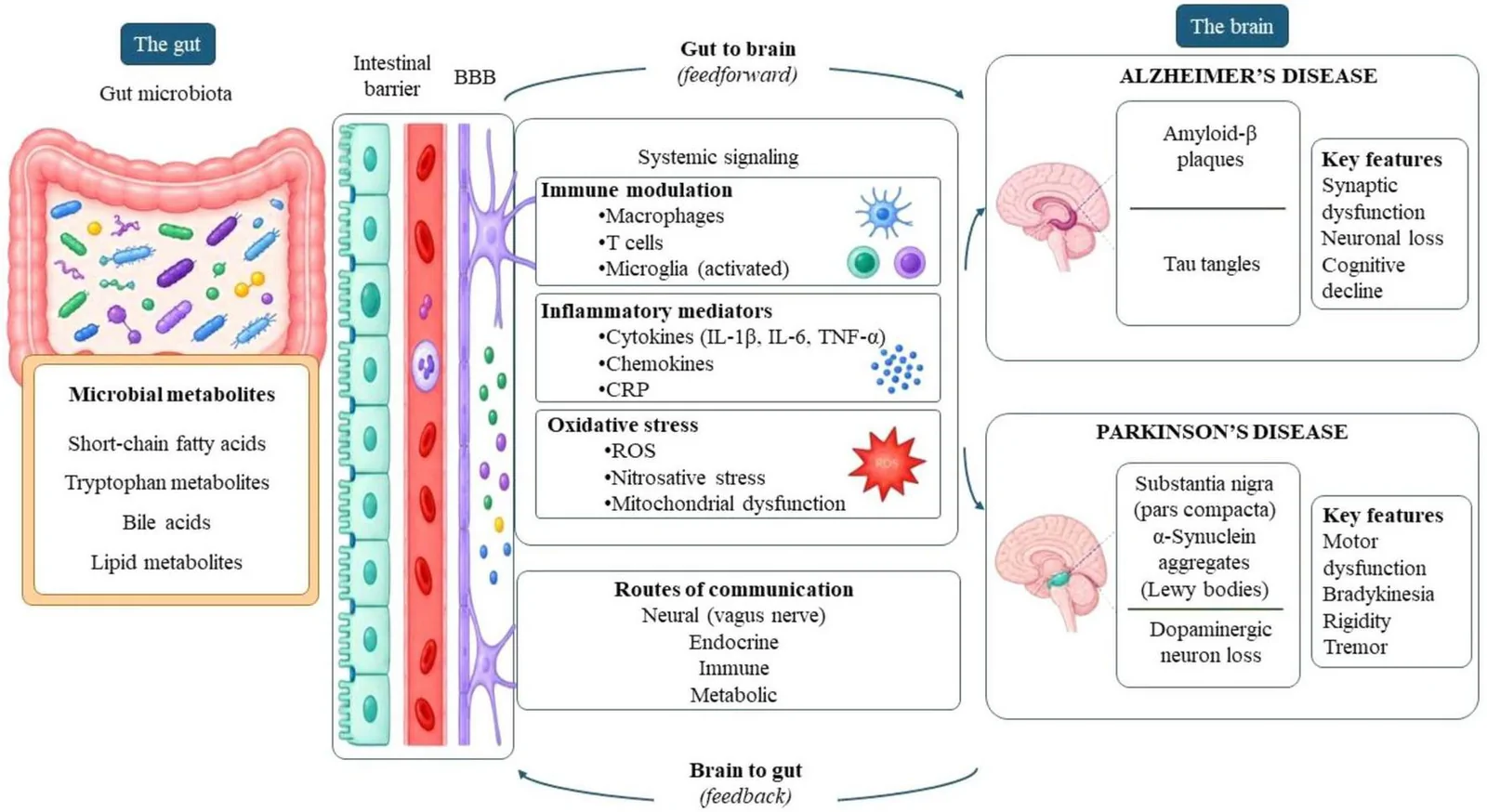
The gut–brain axis in neurodegeneration: a convergent framework. Disruption of gut microbial ecology (dysbiosis) alters the production of microbial metabolites, including short-chain fatty acids (SCFAs), tryptophan-derived metabolites, bile acids, and energy- and lipid-associated metabolites. These metabolic shifts, together with impaired intestinal barrier integrity, shape systemic immune and inflammatory tone, oxidative stress status, and blood–brain barrier (BBB) permeability. Signals arising from this systemic milieu converge on the central nervous system (CNS) and are differentially integrated into disease-specific pathology. In Alzheimer’s disease (AD), these inputs influence amyloid-β accumulation, tau phosphorylation, and the vulnerability of cortical and hippocampal networks, whereas in Parkinson’s disease (PD) they converge on α-synuclein pathology and the selective degeneration of dopaminergic neurons in the substantia nigra pars compacta. The bidirectional arrows indicate that brain-derived signals, including autonomic dysfunction and altered gastrointestinal motility, feed back to further modify gut ecology. This framework positions the microbiome as an upstream regulator of neuroinflammation and neurodegeneration rather than a secondary correlate of disease.

Despite this convergence, cross-disease comparisons remain limited. Most studies examine AD or PD in isolation, obscuring shared pathways, disease-specific signatures, and translationally relevant therapeutic nodes. This review addresses that gap by synthesizing mechanistic evidence across AD and PD to identify common microbiota-dependent processes, delineate disease-selective patterns, and define an integrated ecological–metabolic–neural model of gut–brain axis involvement in neurodegeneration.

## 2 Literature search and study selection

A structured literature search was carried out to gather relevant evidence regarding the contribution of the gut–brain axis, the gut microbiota, microbial metabolites, dietary bioactives, and advanced delivery systems to the pathophysiology and experimental modulation of AD and PD. Because this manuscript was developed as a narrative review, the search and study selection were designed to yield a wide-ranging, mechanistically oriented synthesis, rather than to function as a formal systematic review or meta-analysis.

The literature search was conducted in PubMed/MEDLINE, Scopus, Web of Science, and Google Scholar. It encompassed publications available from January 2010 to August 2026, with a particular focus on recent mechanistic and translational studies. The search terms were organized around four key themes: neurodegenerative disease, the gut–brain axis and microbiota, dietary bioactives, and therapeutic delivery strategies. Representative combinations included: “Alzheimer’s disease” OR “Alzheimer disease” OR “AD,” “Parkinson’s disease” OR “Parkinson disease” OR “PD,” “gut microbiota” OR “gut microbiome” OR “intestinal microbiota,” “gut–brain axis” OR “microbiota–gut–brain axis,” “microbial metabolites” OR “short-chain fatty acids” OR “SCFAs” OR “tryptophan metabolites,” “polyphenols” OR “curcumin” OR “resveratrol” OR “EGCG” OR “anthocyanins” OR “chicoric acid” OR “salvianolic acids,” “synbiotic” OR “probiotic,” and “nanoparticle” OR “nanocarrier” OR “nanodelivery” OR “brain-targeted delivery.” These terms were combined using Boolean operators and adapted to the indexing requirements of individual databases.

The search primarily targeted peer-reviewed primary research articles and relevant review articles that provided mechanistic, biological, or translational insight into interactions among dietary bioactives, gut microbial composition, microbial metabolites, systemic inflammation, metabolic regulation, and CNS outcomes in AD or PD. Priority was given to experimental studies employing established disease models, including APP/PS1 and 5xFAD models of AD, as well as MPTP-, rotenone-, and related models of PD, together with complementary invertebrate models when they offered evidence for microbiome-dependent mechanisms. Studies examining synbiotic interventions, manipulation of the microbiota, microbial metabolite signaling, and nanoparticle-mediated delivery of bioactive compounds were also included when they directly supported the gut–brain axis framework.

Studies were deemed relevant if they covered at least one of the following areas: (1) changes in gut microbiota associated with AD or PD; (2) modulation of gut microbial composition by dietary bioactives or related interventions; (3) alterations in microbial metabolites, especially SCFAs and tryptophan-derived metabolites; (4) mechanistic relationships between intestinal changes and neuroinflammation, oxidative stress, metabolic dysfunction, synaptic disruption, amyloid-β pathology, tau pathology, α-synuclein accumulation, or dopaminergic neuronal damage; (5) findings from microbiota-depletion, supplementation, transplantation, or other microbiota-manipulation studies that support causality; and (6) application of synbiotic or nanoparticle-based approaches to strengthen the biological effects or delivery of neuroprotective compounds.

Studies were excluded if they were unrelated to AD or PD, failed to address the gut–brain axis or a mechanistically relevant biological pathway, or lacked sufficient information to assess the relationship among the intervention, microbiota, metabolites, and neurological outcomes. Exclusion also applied to publications without substantive relevance to the review’s central aims, non-peer-reviewed materials, and studies limited exclusively to unrelated diseases or to non-neurological applications. Reference lists of pertinent articles were reviewed manually to locate additional studies not retrieved through the database searches.

Study selection and evidence synthesis were conducted iteratively. At the outset, titles and abstracts were screened to identify publications that might be relevant to the predefined conceptual domains.

Full-text articles were subsequently evaluated for their mechanistic and translational relevance. Because this was a narrative review, studies were not chosen using a formal systematic-review screening method, and no quantitative meta-analysis or formal risk-of-bias assessment was conducted. Priority was given to studies that offered direct mechanistic support, such as microbiota perturbation, metabolite measurements, pathway-focused analyses, genetic or pharmacological validation, and evaluation of neurological or behavioral outcomes.

The extracted information was arranged by disease, experimental model, intervention, microbial responses, microbial metabolites, molecular signaling pathways, pathological outcomes, and functional or behavioral effects. Emphasis was placed on identifying areas of both agreement and disagreement between AD and PD.

Evidence was integrated to distinguish relatively stable upstream mechanisms, such as microbiome remodeling and metabolite-driven immune and metabolic signaling, from disease-specific downstream processes. In AD, these processes primarily involve amyloid-β and tau pathology. In PD, they include α-synuclein aggregation, dopaminergic neuronal dysfunction, and motor impairment. When the results were not consistent, differences in the experimental model, intervention characteristics, microbial composition, dose, treatment duration, and methodological approaches were taken into account in interpreting the evidence.

## 3 Gut–brain axis in Alzheimer’s disease and Parkinson’s disease: shared and disease-specific pathophysiology

### 3.1 Shared and distinct pathological features of AD and PD

AD and PD are progressive neurodegenerative diseases characterized by different primary neuropathological features, yet they show substantial convergence in upstream mechanisms, including oxidative stress, mitochondrial dysfunction, defective proteostasis, persistent neuroinflammation, synaptic impairment, and metabolic disruption (Fan et al., 2026; Jin et al., 2026a; Phillips and Picard, 2024). In AD, the hallmark pathological changes include extracellular Aβ buildup, tau hyperphosphorylation and aggregation, synaptic failure, and the gradual impairment of cortical and hippocampal network function, ultimately leading to cognitive decline (Behroozi et al., 2026; DeTure and Dickson, 2019). In PD, the key pathological characteristics encompass the misfolding and aggregation of α-synuclein, progressive degeneration of dopaminergic neurons within the substantia nigra pars compacta, dysfunction of mitochondrial and proteostatic systems, and compromised nigrostriatal circuitry, which primarily presents as motor dysfunction (Mingo et al., 2025; Rocha et al., 2018).

Despite these distinctions, AD and PD should not be viewed as mechanistically separate conditions. Both entail prolonged engagement of innate immune signaling pathways, oxidative and mitochondrial stress, disruption of neuronal energy balance, reduced efficiency of cellular clearance mechanisms, and a continuous decline in neuronal resilience (DeTure and Dickson, 2019; Lim and Vendruscolo, 2026; Rocha et al., 2018). These shared processes establish a biological framework in which peripheral influences, such as the intestinal microbiota, microbial metabolites, intestinal epithelial barrier integrity, and systemic immune signaling, may affect the course of disease. Consequently, Aβ/tau pathology in AD and α-synuclein/dopaminergic degeneration in PD reflect disease-specific downstream outcomes of partially overlapping cellular stress and neuroimmune networks, rather than wholly independent pathogenic events.

### 3.2 Gut–brain axis involvement in neurodegenerative pathophysiology

The gut–brain axis is a bidirectional conduit through which gut microbial communities communicate with peripheral tissues and the CNS via microbial metabolites, immune mediators, endocrine signaling, neural pathways, and the modulation of intestinal and blood–brain barrier integrity (Cryan et al., 2019; Morais et al., 2021). Dysbiosis, altered production of microbial metabolites, compromised epithelial barrier function, and increased exposure to microbial components such as endotoxin can drive systemic immune activation and may shape neuroinflammatory pathways (Cattaneo et al., 2017; Quigley, 2017).

In both AD and PD models, changes in gut microbial composition have been linked to reduced microbial diversity, disruption of SCFA-producing populations, expansion of taxa associated with inflammation, and impaired intestinal homeostasis (Romano et al., 2021; Sampson et al., 2016; Vogt et al., 2017). Such shifts can enhance peripheral inflammatory signaling and modify the availability of metabolites that participate in immune regulation, oxidative stress responses, energy metabolism, and neuronal activity (Agus et al., 2018; Dalile et al., 2019) . SCFAs, tryptophan-derived metabolites, bile acids, and other microbial products can modulate epithelial integrity, immune-cell behavior, microglial phenotypes, mitochondrial function, and neuronal signaling, offering potential mechanistic connections between intestinal dysbiosis and CNS vulnerability (Agus et al., 2018; Cryan et al., 2019; Morais et al., 2021). The interconnected relationships among gut dysbiosis, microbial metabolites, systemic signaling, BBB integrity, and disease-specific CNS pathology are illustrated in Figure 1.

### 3.3 Classes of dietary bioactives examined in the gut–brain axis

Within this framework, dietary bioactives can be considered upstream modulators of the gut–brain axis rather than isolated agents acting exclusively on individual neuronal targets (Singh et al., 2024). The present literature primarily encompasses polyphenols and related phytochemicals, including curcumin, resveratrol, quercetin, epigallocatechin-3-gallate (EGCG), anthocyanins, salvianolic acids, chicoric acid, and complex polyphenol-rich preparations (Fakhri et al., 2021; Sun et al., 2023; Zhao et al., 2024). These compounds can influence microbial composition, microbial metabolite production, intestinal barrier function, oxidative stress, and inflammatory signaling, while some may also undergo microbial biotransformation into metabolites with distinct biological activities (Cardona et al., 2013; Ozdal et al., 2016; Tzounis et al., 2011).

A second class comprises microbiota-directed combinations, particularly probiotics, synbiotics, and fecal microbiota transplantation (FMT), which provide more direct approaches for testing whether microbial remodeling contributes causally to neuroprotection (Bonfili et al., 2017; Cryan et al., 2019; Sun et al., 2023). A third class includes engineered delivery systems, particularly nanoparticle-based formulations designed to improve the stability, bioavailability, intestinal exposure, tissue distribution, or brain delivery of poorly bioavailable polyphenols (Anand et al., 2007; Prasad et al., 2014; Yang et al., 2023a). These formulation strategies are mechanistically relevant because altered pharmacokinetics may modify both direct host signaling and the extent to which compounds interact with the intestinal microbiome (Fakhri et al., 2021; Singh et al., 2024).

## 4 Gut–brain axis in Alzheimer’s disease

Emerging preclinical evidence consistently demonstrates that AD pathology is tightly linked to gut microbiota dysregulation, impaired microbial metabolite production, and systemic inflammatory signaling along the gut–brain axis (Krishaa et al., 2023; Liu et al., 2020). Across multiple rodent and invertebrate models, dietary polyphenols, synbiotic formulations, and nano-delivery systems converge on a shared mechanistic pattern involving microbiota restoration, reduced amyloid burden, and improved cognitive performance.

### 4.1 Microbiota remodeling as a central therapeutic node

A consistent finding across studies is that AD-like pathology is associated with microbial imbalance characterized by enrichment of pro-inflammatory taxa and depletion of beneficial bacteria. In APP/PS1 mice, multitarget interventions such as curcumin analog combined with anti-Aβ immunotherapy restored gut microbial composition toward wild-type levels, accompanied by reduced amyloid burden and improved behavioral outcomes (Su et al., 2022). Similarly, synbiotic formulations containing probiotics and polyphenols normalized microbial diversity and improved metabolic stability in AD Drosophila models, leading to reduced Aβ toxicity and enhanced survival (Westfall et al., 2019). The latter findings should be interpreted as invertebrate evidence for microbiota-dependent modulation rather than direct evidence of conserved mammalian AD mechanisms.

Resveratrol-based nanodelivery systems further reinforce this microbiota-dependent mechanism. Brain-targeted resveratrol-loaded chitosan nanoparticles significantly reduced obesity-related AD pathology while reshaping gut microbiota, characterized by decreased abundance of *Helicobacter*, *Colidextribacter*, and *Parasutterella*, and increased levels of beneficial taxa such as *Bifidobacterium* and *Allobaculum* (Yang et al., 2023a). These microbial shifts were tightly associated with improvements in insulin resistance and reduced amyloid and tau pathology via the JNK/AKT/GSK3β signaling axis (Yang et al., 2023a).

### 4.2 Metabolic and microbial metabolite pathways

A key mechanistic hallmark across AD models is the restoration of metabolic homeostasis and SCFA production (Zhu et al., 2020). Curcumin administration in AD-like metabolic models restored gut microbiota balance and normalized SCFA levels, which correlated with reduced oxidative stress and improved cognitive outcomes through NRF2-mediated antioxidant signaling (Lamichhane et al., 2024; Wang, 2026). Similarly, resveratrol nanoparticles improved lipid- and insulin-associated microbial profiles, linking gut microbial shifts to systemic metabolic regulation via AKT/GSK3β signaling (Yang et al., 2023a). These findings should be interpreted within the broader AD pathophysiological framework established above: metabolic regulation represents an important disease-modifying interface, but it does not exclude inflammatory, oxidative, mitochondrial, or proteostatic mechanisms that are also shared with PD.

### 4.3 Neuroimmune modulation and amyloid clearance

Neuroimmune regulation represents a central convergence point in gut–brain axis research on AD, particularly in relation to amyloid-β clearance and synaptic protection (Aranda-Abreu et al., 2026; Müller and Di Benedetto, 2025). In APP/PS1 mouse models, combination therapy using a curcumin analog alongside an anti-Aβ antibody significantly reduced pro-inflammatory cytokine production while enhancing microglial phagocytic activity, leading to a marked decrease in Aβ burden and measurable improvements in nesting and cognitive-like behaviors (Su et al., 2022). Importantly, these neuroimmune benefits were accompanied by normalization of gut microbiota composition, indicating that restoration of microbial homeostasis may actively contribute to central immune regulation and facilitate amyloid clearance rather than acting as a peripheral correlate alone.

Consistent with these findings, a broad range of polyphenol-based interventions, including curcumin, resveratrol, quercetin, and anthocyanins, have been shown to exert convergent anti-inflammatory and antioxidant effects across AD models (Davinelli et al., 2025; Lv et al., 2018; Upadhyay et al., 2024; Wu et al., 2025). Importantly, NF-κB and NRF2 signaling should not be regarded as AD-specific pathways. Both are also implicated in PD, illustrating the mechanistic overlap between the two disorders despite their different dominant neuropathological targets.

### 4.4 Neurotrophic signaling and cognitive enhancement

Beyond the well-established roles of amyloid reduction and neuroinflammation control, emerging evidence highlights neurotrophic signaling as a critical downstream mechanism linking microbiota modulation to cognitive improvement in AD (Gao et al., 2022). In APP/PS1 mouse models, quercetin administration was shown to increase gut microbial diversity while selectively enriching taxa such as *Glutamicibacter* and *Facklamia* (Lei et al., 2025). These microbial shifts were closely associated with upregulation of BDNF expression and modulation of microRNA-related regulatory networks, ultimately leading to improved memory performance and reduced amyloid-β deposition (Lv et al., 2018). This suggests that microbiota-driven changes may directly influence synaptic plasticity and neuronal resilience through neurotrophin-dependent pathways.

Likewise, in Drosophila melanogaster models of AD, salvianolic acids were found to alter gut microbial composition by reducing *Enterococcus* and increasing *Lactiplantibacillus*, which was associated with better metabolic homeostasis and a longer lifespan (Go et al., 2024). These results offer valuable causal and mechanistic support for host–microbiota interactions, yet they should not be taken as direct evidence that the same BDNF-dependent cognitive mechanisms function in mammalian AD. Substantial differences in intestinal anatomy, microbial ecology, immune organization, nervous-system structure, metabolism, and lifespan restrict straightforward extrapolation. Therefore, in this review, Drosophila work is treated as complementary, hypothesis-generating evidence, while mammalian studies form the primary foundation for disease-specific interpretation.

### 4.5 Microbial metabolite transformation and bioactivity amplification

An additional mechanistic layer in gut–brain axis–mediated neuroprotection involves the microbial biotransformation of dietary polyphenols into smaller, more bioactive metabolites with enhanced physiological activity (Nazzaro et al., 2025). *In vitro* digestion and fermentation studies using marine polyphenols derived from Sargassum wightii demonstrated that gut microbial processing generates low-molecular-weight metabolites with significantly increased antioxidant capacity and acetylcholinesterase inhibitory activity (Gade and Kumar, 2025). This observation is particularly relevant to the conceptual framework of the review because it demonstrates that the microbiome can modify the biological activity of the intervention itself, rather than merely responding to treatment.

### 4.6 Integrated mechanistic interpretation

Therefore, the AD evidence supports a multidimensional model rather than one that is exclusively metabolic–amyloid (Cattaneo et al., 2017; Morais et al., 2021; Vogt et al., 2017). Remodeling of the microbiota can affect short-chain fatty acid production, metabolic homeostasis, intestinal and systemic inflammation, oxidative stress, neurotrophic signaling, and immune regulation (Agus et al., 2018; Cryan et al., 2019; Dalile et al., 2019). These processes may then interact with AD-relevant pathways that include Aβ processing, tau phosphorylation, synaptic plasticity, and microglial function (Cattaneo et al., 2017; De Strooper and Karran, 2016; DeTure and Dickson, 2019). Several underlying mechanisms, including NF-κB activation, NRF2 signaling, mitochondrial dysfunction, oxidative stress, short-chain fatty acid signaling, and impaired barrier integrity, are also implicated in PD (Cryan et al., 2019; Morais et al., 2021; Sampson et al., 2016). Accordingly, the distinguishing feature of AD is not merely the exclusive presence of metabolic or amyloid-related mechanisms, but rather their more robust integration with Aβ/tau pathology, insulin resistance, lipid dysregulation, and vulnerability of cognitive networks (Arnold et al., 2018; Bloom, 2014; DeTure and Dickson, 2019).

## 5 Gut–brain axis in Parkinson’s disease

Emerging preclinical evidence strongly supports the role of the microbiota–gut–brain axis as a mechanistic bridge linking intestinal dysbiosis, systemic inflammation, and dopaminergic neurodegeneration in PD (Guerrero-Torres et al., 2026). Across rodent, fly, and translational models, dietary polyphenols (curcumin, resveratrol, EGCG, anthocyanins, chicoric acid) and nanoparticle-based formulations consistently demonstrate neuroprotective efficacy mediated through microbiome remodeling, restoration of microbial metabolites (notably SCFAs), and suppression of gut-derived inflammatory signaling (Fakhri et al., 2021; Sampson et al., 2016; Sun et al., 2023). These convergent findings suggest that targeting gut microbial ecosystems may represent a unifying therapeutic strategy for PD pathophysiology (Cryan et al., 2019; Morais et al., 2021).

### 5.1 Polyphenols and microbiota-driven neuroprotection

In MPTP/P-induced PD mouse models, resveratrol reshaped gut microbial ecology, increased Prevotellaceae, Rikenellaceae, *Blautia*, and *Alistipes*, normalized the Firmicutes/Bacteroidetes ratio, and reduced TNF-α, IL-6, and IL-1β (Tao et al., 2023). These changes were associated with preservation of dopaminergic neurons and improved motor and gastrointestinal outcomes. Chicoric acid and anthocyanins similarly modified microbial composition, restored SCFA-associated microbial functionality, and reduced inflammatory and glial responses (Cao et al., 2024; Wang et al., 2022). These results indicate a significant contribution of gut-derived inflammatory signaling to PD, yet they should not be taken as proof that PD is solely an immune-inflammatory condition. Metabolic dysregulation, oxidative stress, mitochondrial dysfunction, defective proteostasis, α-synuclein aggregation, and neuronal survival pathways substantially overlap with mechanisms reported in AD.

### 5.2 Curcumin and gut–brain axis modulation in PD

Curcumin administration in MPTP-induced mice improved motor dysfunction and dopaminergic neuronal survival while correcting intestinal dysbiosis. Reductions in Enterobacteriaceae and Enterococcaceae and improvements in intestinal barrier integrity were associated with activation of SIRT1/NRF2 and suppression of AIM2/caspase-1-associated inflammatory pyroptosis (Zhong et al., 2022). Curcumin also normalized SCFA profiles and carbohydrate metabolism, linking microbial metabolic restoration with reduced neuroinflammation and dopaminergic preservation (Cai et al., 2023; Cao et al., 2024). The involvement of SCFA signaling, NRF2, Akt, oxidative stress, and metabolic regulation illustrates that PD shares several mechanistic nodes with AD, even though their downstream neuronal vulnerabilities differ.

### 5.3 Resveratrol and microbiota–metabolic interactions

Resveratrol reshaped gut microbial composition and reduced systemic inflammation in MPTP/P-induced PD, while FMT from treated animals reproduced improvements in motor function and dopaminergic neuronal survival (Tao et al., 2023). Brain-targeted resveratrol nanoparticles additionally modified microbial communities and insulin-related signaling through the JNK/AKT/GSK3β pathway (Yang et al., 2023a). The latter finding is particularly relevant to the cross-disease comparison because JNK/AKT/GSK3β signaling is often emphasized in AD but can also participate in PD-associated neuronal survival, metabolic regulation, and stress responses. Thus, pathway involvement should be interpreted according to disease context rather than assigned exclusively to one disorder.

### 5.4 EGCG, synbiotics, and microbiota-dependent signaling pathways

Mechanistic evidence from Drosophila provides causal support for microbiota dependence. Xu et al. (2020) demonstrated that EGCG improved dopaminergic dysfunction in PINK1-mutant Drosophila melanogaster, whereas antibiotic-mediated microbiota depletion abolished this effect. Transcriptomic analysis identified TotM as a candidate host mediator. However, TotM signaling and the Drosophila nervous system cannot be considered direct equivalents of mammalian PD pathways. The principal value of this model is its experimental demonstration that microbiota integrity can be necessary for a bioactive compound’s phenotype, while the molecular mechanisms identified require validation in mammalian systems (Xu et al., 2020).

In a complementary AD model, Westfall et al. (2019) demonstrated that a polyphenol-rich synbiotic formulation significantly extended lifespan and improved locomotor performance in Drosophila melanogaster expressing human Aβ pathology. These effects were linked to coordinated gut–brain axis modulation, including enhanced microbial metabolic stability, reduced oxidative stress, and improved mitochondrial function. Mechanistically, activation of PPARγ signaling emerged as a central node integrating microbial and host metabolic responses, further reinforcing the concept that synbiotic interventions exert neuroprotective effects through conserved immunometabolic pathways. Nevertheless, these results must be considered in light of the biological context of the Drosophila model. Variations in intestinal anatomy, microbial composition, immune organization, nervous-system architecture, metabolism, and lifespan restrict the ability to extrapolate the observed effects directly to mammalian AD. In particular, the enhanced lifespan and locomotor performance seen in *Drosophila* do not, by themselves, demonstrate equivalent effects on mammalian cognition or AD-specific neuropathology (Westfall et al., 2019).

Taken together, these invertebrate studies offer complementary causal evidence that the integrity of the microbiota can be functionally important for neuroprotection mediated by polyphenols and synbiotics. Their main contribution is to bolster the mechanistic inference that host–microbiota interactions can drive the biological activity of dietary bioactive compounds. However, these findings should be distinguished from evidence in mammals and should be regarded as hypothesis-generating for mammalian gut–brain mechanisms, rather than as direct confirmation that conserved AD or PD pathways are involved. Confirmation in mammalian models is still required to determine the relevance of these mechanisms to mammalian neurodegeneration and, ultimately, human disease.

### 5.5 Integrated interpretation of PD mechanisms

Across the PD literature, a highly consistent integrated mechanistic framework emerges in which multiple dietary bioactives converge on a limited set of gut–brain regulatory axes. A first dominant axis involves inflammation suppression, where interventions such as curcumin, resveratrol, chicoric acid, and anthocyanins inhibit canonical immune signaling pathways including TLR4/MyD88/NF-κB activation, SIRT1/NRF2 redox regulation, and inflammasome-associated pyroptosis (e.g., AIM2/caspase-1), thereby reducing systemic and intestinal pro-inflammatory tone (Tao et al., 2023; Wang et al., 2022; Zhong et al., 2022). A second recurring mechanism is metabolic restoration, particularly normalization of SCFA production and broader carbohydrate and lipid metabolic networks, which collectively support epithelial integrity and energy homeostasis and indirectly influence neuroimmune balance (Cai et al., 2023; Cao et al., 2024). In parallel, nearly all interventions report microbial community rebalancing, typically characterized by increased abundance of beneficial taxa such as Lactobacillus, Bifidobacterium, Blautia, Alistipes, and SCFA-producing organisms, alongside a reduction in dysbiosis-associated groups including Proteobacteria and Enterobacteriaceae, as well as disease-enriched taxa such as Helicobacter in metabolic contexts (Tao et al., 2023; Yang et al., 2023a; Zhong et al., 2022). These microbial shifts are tightly coupled with activation of neuronal survival pathways, particularly Akt-mediated anti-apoptotic signaling and downstream cytoprotective cascades, which contribute to dopaminergic neuron preservation in MPTP and rotenone models (Mu et al., 2026). Importantly, these gut-derived effects are reinforced by improvements in gut barrier integrity and mucosal immune regulation, limiting translocation of inflammatory mediators and microbial endotoxins into systemic circulation. Collectively, these converging lines of evidence support a unified model in which polyphenols and related bioactives exert neuroprotective effects in PD primarily through restoration of gut microbial homeostasis, with downstream modulation of host inflammatory, metabolic, and neuronal survival signaling networks forming the mechanistic bridge between the intestine and the brain.

## 6 Comparative gut–brain axis mechanisms in Alzheimer’s disease and Parkinson’s disease

Across recent preclinical literature, AD and PD share a convergent mechanistic framework centered on gut microbiota dysbiosis, microbial metabolite signaling, and neuroinflammation, yet they diverge in disease-specific pathological targets and dominant systemic interactions. A comparative synthesis of these mechanisms highlights both shared gut–brain axis pathways and distinct neurobiological endpoints.

### 6.1 Core pathological divergence shapes gut–brain signaling profiles

In AD models, the primary pathological drivers are Aβ accumulation and tau hyperphosphorylation, as demonstrated in APP/PS1, 5xFAD, and obesity-linked AD models (Su et al., 2022; Upadhyay et al., 2024; Yang et al., 2023a). In contrast, PD models such as MPTP-, rotenone-, and PINK1-based systems predominantly exhibit dopaminergic neuronal degeneration in the substantia nigra, leading to motor dysfunction (Tao et al., 2023; Xu et al., 2020; Zhong et al., 2022). These disease-specific pathological and neuroanatomical vulnerabilities set apart the principal downstream consequences of AD and PD, yet they should not be taken as support for entirely distinct gut–brain mechanisms. Both conditions show marked overlap in upstream processes, including gut microbiota dysbiosis, changes in microbial metabolite production, intestinal and blood–brain barrier dysfunction, systemic inflammation, oxidative stress, mitochondrial dysfunction, and microglial activation (Cryan et al., 2019; Morais et al., 2021). Therefore, the gut–brain axis may be regarded as a common upstream regulatory framework that is subsequently modified by disease-specific molecular and neuronal vulnerabilities.

In AD, interventions frequently emphasize attenuation of Aβ/tau pathology, restoration of metabolic and insulin signaling, and preservation of hippocampal and cortical function (Arnold et al., 2018; DeTure and Dickson, 2019). In PD, interventions more often emphasize suppression of gut-derived inflammatory signaling, protection of dopaminergic neurons, mitochondrial homeostasis, and preservation of nigrostriatal function (Morais et al., 2021; Sampson et al., 2016). However, these differences reflect differences in mechanistic emphasis rather than pathways that exclude each other. Metabolic dysfunction, oxidative stress, neuroinflammation, and disrupted cellular energy homeostasis also play roles in PD, while inflammatory, mitochondrial, and oxidative mechanisms are core to AD pathogenesis (De Strooper and Karran, 2016; Morais et al., 2021). Therefore, the distinct pathological end points of AD and PD result from how shared gut-derived immunometabolic signals interact with disease-specific molecular features and neuroanatomical susceptibilities.

### 6.2 Microbiome remodeling: anti-amyloid vs anti-inflammatory signatures

Microbiome remodeling represents a central convergent mechanism in both AD and PD, yet the resulting ecological signatures differ in a disease-specific manner. In AD models, microbial interventions consistently drive a pro-anti-amyloid and metabolically stabilizing microbiome profile (Vogt et al., 2017; Yang et al., 2023a). This is characterized by an increased abundance of beneficial taxa such as *Bifidobacterium*, *Allobaculum*, and other SCFA-producing bacteria, alongside a restoration of overall microbial diversity and ecological balance (Vogt et al., 2017; Yang et al., 2023a). Concurrently, there is a marked reduction in inflammation- and obesity-associated taxa, including Helicobacter and Parasutterella (Vogt et al., 2017; Yang et al., 2023a). These microbial shifts are closely linked to improved lipid metabolism, enhanced systemic metabolic homeostasis, and a reduction in amyloidogenic processes, collectively contributing to decreased amyloid-β burden and improved neurocognitive outcomes.

In contrast, PD models exhibit a microbiome remodeling pattern that is more strongly oriented toward anti-inflammatory and neuroprotective immune balance rather than metabolic correction alone. This includes increased abundance of taxa such as Prevotellaceae, Rikenellaceae, *Blautia*, and *Alistipes* (Tao et al., 2023), along with restoration of SCFA-producing and mucosal-protective bacterial populations. At the same time, there is a consistent reduction in pro-inflammatory bacterial families, particularly Enterobacteriaceae, which are strongly associated with gut-derived endotoxemia and systemic immune activation (Wang et al., 2022; Zhong et al., 2022). Collectively, these findings indicate that while both AD and PD interventions converge on microbial normalization, the functional direction of remodeling differs: AD is predominantly characterized by metabolic–amyloid regulation, whereas PD is primarily defined by immune–inflammatory stabilization.

The key preclinical studies examining microbiome modulation and gut–brain signaling in models of AD are summarized in Table 1, which emphasizes the links among dietary bioactive compounds, microbial reorganization, molecular pathways, and neurocognitive outcomes.

**TABLE 1:** Integrated preclinical evidence of gut–brain axis modulation in Alzheimer’s disease models.

References	Model	Intervention	Microbiome/metabolite effects	Key pathways	Core functional outcomes
Su et al., 2022	APP/PS1 mice	Curcumin analog + anti-Aβ antibody	Normalized gut microbiota composition vs. APP/PS1 controls	Microglial phagocytosis; inflammatory cytokine reduction	↓ Aβ burden; improved nesting behavior
Yang et al., 2023a	Obesity-related AD mice	Brain-targeted resveratrol-CS/TPP nanoparticles	↓ *Helicobacter*, *Colidextribacter*, *Parasutterella*; ↑ *Bifidobacterium*, *Allobaculum*	JNK/AKT/GSK3β; insulin resistance axis	↓ Aβ and Tau phosphorylation; improved cognition
Lamichhane et al., 2024	3×Tg-AD mice	Curcumin	↑ Beneficial microbiota	Liver–gut–brain axis	Improved spatial memory and cognitive
Mu et al., 2026	Neurodegeneration model (AD-relevant pathway)	Curcumin nanoparticles	Microbiota remodeling; ↑ antioxidant-associated taxa	Akt signaling; anti-apoptosis	Neuroprotection; improved brain function
Westfall et al., 2019	AD Drosophila (Aβ42)	Synbiotic (probiotics + polyphenols)	↑ Beneficial microbiota; metabolic stabilization	PPARγ; oxidative stress; mitochondrial regulation	Improved survival; ↓ Aβ toxicity; improved motility
Upadhyay et al., 2024	APP/PS1 + 5×FAD mice	Dual-dose Triphala	↑ *Bacteroides*, Proteobacteria balance; microbiota restructuring	APP pathway modulation; inflammation	Improved cognition; reduced neuroinflammation
Lv et al., 2018	APP/PS1 mice	Quercetin	↑ Microbial diversity; enrichment of *Glutamicibacter*, *Facklamia*	BDNF upregulation; miRNA modulation	Reduced Aβ plaques; improved memory
Go et al., 2024	Aβ42 Drosophila	Salvianolic acids	↓ *Corynebacterium*, *Enterococcus*; ↑ *Lactiplantibacillus*	Urea cycle; metabolic regulation	Extended lifespan; improved neuroprotection
Gade and Kumar, 2025	*In vitro* fermentation + digestion model	Marine polyphenols	Enhanced microbial biotransformation → LMW metabolites; ↑ antioxidant capacity	AChE inhibition; metal chelation	Potential anti-AD bioactivity
Yang et al., 2023a	Obesity-linked AD mice	Resveratrol nanoparticles	Gut microbiota normalization; lipid metabolism-associated taxa modulation	JNK/AKT/GSK3β	↓ Insulin resistance; ↓ Aβ aggregation

Additional findings from PD models indicate a gut–brain regulatory profile that is only partially overlapping with that in AD, but is more pronounced in its immune–inflammatory features. This profile is marked by the reestablishment of microbial homeostasis, increased short-chain fatty acid production, improved intestinal barrier function, and the inhibition of gut-derived inflammatory signaling (Table 2).

**TABLE 2:** Integrated preclinical evidence of gut–brain axis modulation in Parkinson’s disease models.

References	Model	Intervention	Microbiome/metabolite effects	Key pathways	Core functional outcomes
Tao et al., 2023	MPTP/P chronic mice	Resveratrol (oral)	↑ Prevotellaceae, Rikenellaceae, *Blautia*, *Alistipes*; ↓ F/B ratio; restored gut diversity; ↓ colonic TNF-α, IL-6, IL-1β	Gut–brain axis; inflammation suppression; dopaminergic protection	Improved motor function; ↑ TH neurons; reduced GI dysfunction
Zhong et al., 2022	MPTP mice	Curcumin	↓ Enterobacteriaceae, Enterococcaceae; restored microbial balance; improved gut barrier	SIRT1/NRF2; pyroptosis inhibition	Improved motor deficits; protection of dopaminergic neurons
Cai et al., 2023	MPTP mice	Curcumin	Restored SCFAs; reduced dysbiosis; improved carbohydrate metabolism	SCFA signaling; gut–brain axis	Reduced neuroinflammation; neuroprotection
Mu et al., 2026	MPTP mice	Curcumin nanoparticles	Gut microbiota remodeling; improved antioxidant profile	Akt signaling; anti-apoptosis	Improved motor behavior; neuroprotection
Wang et al., 2022	MPTP mice	Chicoric acid	↓ Bacteroidetes shift; ↑ *Lactobacillus*, *Ruminiclostridium*; restored SCFAs	TLR4/MyD88/NF-κB	Reduced neuroinflammation; dopaminergic neuron protection
Cao et al., 2024	MPTP mice	*Lycium ruthenicum* anthocyanins	↓ Coriobacteriaceae, Parvibacter; ↑ beneficial SCFA-producing taxa	Anti-inflammatory microbial axis	Reduced motor deficits; ↑ dopamine neuron survival
Xu et al., 2020	PINK1 *Drosophila* PD model	EGCG	Restored microbiota composition; EGCG effect lost under microbiota depletion	TotM signaling pathway	Improved locomotion; rescued dopaminergic defects
Katunina et al., 2024	PD conceptual model	Polyphenols (curcumin focus)	Gut dysbiosis reduction; anti-inflammatory microbiome shift	Microbiota–gut–brain axis	Reduced neurodegenerative triggers

### 6.3 Metabolite convergence: SCFAs as shared signaling hubs

Microbiome remodeling is a key convergent mechanism shared by both AD and PD, marked by substantial overlap in restoring microbial diversity, enriching beneficial and SCFA-associated taxa, and reducing microbial groups linked to dysbiosis. In AD models, microbiome-targeted interventions have been reported to yield a metabolically stabilizing and neuroprotective microbial profile, defined by increased abundance of taxa such as Bifidobacterium, Allobaculum, and other SCFA-producing bacteria, along with recovery of overall microbial diversity and ecological balance. At the same time, decreases in inflammation- and obesity-associated taxa, including Helicobacter and Parasutterella, have also been observed (Yang et al., 2023a). These microbial shifts are linked to better lipid- and insulin-related metabolic homeostasis and reduced amyloidogenic processes, thereby lowering amyloid-β burden and supporting improved neurocognitive outcomes. Nonetheless, these findings should not be viewed as exclusively anti-amyloid, since inflammatory signaling, oxidative stress, intestinal barrier dysfunction, and immune regulation are also integral elements of AD pathology and may underlie the neuroprotective effects that have been observed.

In PD models, microbiome remodeling similarly involves restoration of microbial homeostasis, including increased abundance of taxa such as Prevotellaceae, Rikenellaceae, *Blautia*, and *Alistipes*, together with recovery of SCFA-producing and mucosal-protective bacterial populations. Reductions in dysbiosis-associated and potentially pro-inflammatory groups, including Enterobacteriaceae, have also been reported (Cai et al., 2023; Tao et al., 2023). These changes are linked to improved intestinal barrier function, reduced gut-derived inflammatory signaling, and preservation of dopaminergic neurons. Nevertheless, PD should not be viewed as solely an immune–inflammatory disorder. Metabolic dysfunction, oxidative stress, mitochondrial dysfunction, and changes in microbial metabolite signaling also shape PD pathophysiology and engage with inflammatory pathways (Morais et al., 2021).

Accordingly, the current body of evidence is consistent with a framework of shared ecological normalization rather than a rigid “anti-amyloid versus anti-inflammatory” split. Interventions targeting both AD and PD can restructure the gut microbiota in ways that affect SCFA production, barrier integrity, systemic inflammation, oxidative stress, and host metabolism (Cryan et al., 2019; Morais et al., 2021). The main distinction is the relative downstream emphasis and the disease-specific interpretation of these overlapping signals: metabolic and insulin-related pathways may be especially pertinent to amyloid and tau pathology in AD, while inflammatory and mitochondrial pathways may be particularly important for α-synuclein-associated dopaminergic neurodegeneration in PD (DeTure and Dickson, 2019; Morais et al., 2021). Overall, these constitute intersecting mechanistic networks with different, disease-specific susceptibilities rather than separate, independent pathways.

The principal preclinical studies investigating microbiome modulation and gut–brain signaling in AD models are summarized in Table 1, highlighting the relationships among dietary bioactives, microbial remodeling, molecular pathways, and neurocognitive outcomes.

Complementary evidence from PD models demonstrates a partially overlapping pattern of gut–brain regulation characterized by restoration of microbial homeostasis, SCFA production, intestinal barrier function, metabolic balance, and suppression of gut-derived inflammatory signaling (Table 2). Taken together, Tables 1, 2 illustrate that microbial remodeling provides a shared upstream interface between AD and PD, whereas the subsequent metabolic, immune, and neuronal responses are shaped by disease-specific pathological and neuroanatomical contexts.

### 6.4 Pathway-level differences: metabolic vs. inflammatory axis dominance

At the intracellular signaling level, microbiota-modulating interventions reveal distinct but partially overlapping pathway architectures that differentiate AD from PD. In AD models, the dominant signaling networks include AKT/GSK3β pathways that regulate amyloid-β production and tau phosphorylation, NF-κB-mediated neuroinflammatory cascades, NRF2-dependent antioxidant defense mechanisms, and BDNF-associated synaptic plasticity and neuronal resilience (Su et al., 2022; Westfall et al., 2019; Yang et al., 2023a). These pathways interact rather than operate independently, linking metabolic dysfunction, oxidative stress, neuroinflammation, and proteinopathy. Thus, although metabolic and amyloid-related mechanisms are prominent in AD, inflammatory and redox pathways are also integral to disease progression and are modulated by gut-derived signals.

In contrast, PD models are characterized by a stronger emphasis on inflammatory and stress-response signaling networks originating from the gut–brain axis. Key pathways include the SIRT1/NRF2 axis, which regulates oxidative stress and mitochondrial homeostasis in both intestinal and neural tissues, the TLR4/MyD88/NF-κB inflammatory cascade that mediates gut-derived immune activation, and pyroptosis-associated mechanisms such as AIM2/caspase-1 signaling that amplify neuroinflammatory cell death. In addition, Akt-mediated neuronal survival pathways contribute to dopaminergic neuron resilience under inflammatory stress conditions (Mu et al., 2026; Wang et al., 2022; Zhong et al., 2022). Importantly, these pathways are not exclusive to PD. NRF2, NF-κB, Akt, mitochondrial dysfunction, oxidative stress, and inflammatory signaling are also relevant to AD, whereas metabolic dysfunction and disrupted cellular energy homeostasis may play a role in PD pathogenesis.

Accordingly, the differentiation between AD and PD should be understood in terms of relative pathway dominance and disease-specific downstream susceptibility, rather than relying on distinct signaling architectures (Cryan et al., 2019; Morais et al., 2021). Shared gut-derived metabolic and immune signals can converge on common intracellular nodes, including Akt, NRF2, NF-κB, and mitochondrial stress pathways; however, the resulting effects are determined by the prevailing pathological context (Cattaneo et al., 2017; De Strooper and Karran, 2016). In AD, these interactions tend to drive amyloid-β accumulation, tau hyperphosphorylation, synaptic dysfunction, and cortical–hippocampal vulnerability (Bloom, 2014; DeTure and Dickson, 2019). In PD, the same broader immunometabolic and oxidative-stress networks interact with α-synuclein pathology and mitochondrial dysfunction to promote selective vulnerability of dopaminergic neurons in the substantia nigra (Morais et al., 2021; Sampson et al., 2016). This overlapping yet disease-contextualized pathway architecture provides a more accurate representation of gut–brain signaling in neurodegeneration than a strict metabolic–amyloid versus inflammatory dichotomy (Cryan et al., 2019; Morais et al., 2021).

### 6.5 Systemic disease coupling: metabolic vs. immune axis dominance

A key distinction between AD and PD lies in the broader systemic context that drives gut–brain axis dysfunction. AD is more strongly associated with metabolic dysregulation, particularly insulin resistance, obesity-related inflammation, and impaired lipid metabolism. This is consistently observed in obesity-linked AD models, where interventions such as resveratrol and curcumin restore glucose homeostasis, improve systemic metabolic balance, and concurrently reduce amyloid-β accumulation and related neurodegenerative pathology (Yang et al., 2023a). However, these metabolic effects are closely interconnected with inflammatory and redox pathways, including NF-κB and NRF2 signaling, indicating that metabolic dysfunction in AD cannot be considered independently of immune regulation.

In contrast, PD is more tightly coupled to immune–gut axis dysfunction, in which intestinal inflammation, compromised epithelial barrier integrity, and microbial endotoxin translocation act as primary triggers of neuroinflammatory cascades and dopaminergic neuron degeneration (Tao et al., 2023; Zhong et al., 2022). Pathways such as TLR4/MyD88/NF-κB and SIRT1/NRF2 therefore serve as points where immune and metabolic stress intersect, rather than reflecting inflammatory mechanisms that are exclusively specific to PD (Morais et al., 2021; Sampson et al., 2016). Similarly, SCFA-dependent signaling, antioxidant responses, and Akt-mediated neuronal survival pathways are relevant to both conditions, even though the downstream outcomes may vary depending on the neural circuits involved and the underlying pathological substrates (Cattaneo et al., 2017; Cryan et al., 2019; Morais et al., 2021).

Accordingly, AD and PD should not be portrayed as purely metabolic versus immune-inflammatory disorders. Instead, both conditions feature an integrated metabolic–immune–redox network in which the relative contribution of individual processes differs (De Strooper and Karran, 2016; Morais et al., 2021). AD models more prominently connect insulin resistance and lipid dysregulation to amyloid and tau pathology, whereas PD models more strongly highlight intestinal inflammation, barrier dysfunction, mitochondrial stress, and dopaminergic vulnerability (Cattaneo et al., 2017; Morais et al., 2021; Sampson et al., 2016; Vogt et al., 2017). This refined distinction maintains disorder-specific features while acknowledging substantial mechanistic overlap, and it supports a framework in which gut-derived metabolic and immune signals jointly influence neurodegenerative susceptibility in both diseases (Cryan et al., 2019; Morais et al., 2021).

### 6.6 Functional outcomes: cognition versus motor control

The disease-specific organization of these pathways ultimately produces distinct functional outcomes (DeTure and Dickson, 2019; Morais et al., 2021). In AD models, microbiome-targeted interventions are primarily associated with improvements in learning and memory, reduced Aβ burden, attenuation of synaptic dysfunction, and preservation of hippocampal and cortical function (Cattaneo et al., 2017; Vogt et al., 2017). In PD models, comparable interventions more frequently improve motor performance, preserve substantia nigra dopaminergic neurons, and ameliorate gastrointestinal dysfunction associated with impaired enteric and autonomic regulation (Morais et al., 2021; Sampson et al., 2016). Despite these divergent clinical endpoints, a shared outcome across both disease groups is a consistent reduction in neuroinflammation and an overall enhancement of neural resilience, underscoring the gut microbiota as a common upstream regulator of brain health in neurodegeneration (Cryan et al., 2019; Morais et al., 2021).

### 6.7 Integrated interpretation

The comparative evidence supports a model in which the gut–brain axis constitutes a shared upstream regulatory system rather than a disease-specific pathway (Cryan et al., 2019; Morais et al., 2021). Microbial remodeling, SCFA and tryptophan metabolism, intestinal barrier regulation, systemic immune signaling, oxidative stress, and mitochondrial homeostasis provide common mechanistic interfaces between the gastrointestinal tract and CNS (Cattaneo et al., 2017; Sampson et al., 2016; Vogt et al., 2017). Disease specificity emerges downstream, where these signals interact with distinct pathological substrates and vulnerable neural circuits.

In AD, the dominant downstream organization links metabolic dysfunction, insulin signaling, AKT/GSK3β, NRF2, NF-κB, and related pathways to Aβ/tau pathology and cortical–hippocampal dysfunction (Cattaneo et al., 2017; De Strooper and Karran, 2016; DeTure and Dickson, 2019). In PD, gut-derived inflammatory and metabolic signals more prominently interact with TLR4/MyD88/NF-κB, SIRT1/NRF2, mitochondrial stress, α-synuclein pathology, and selective dopaminergic vulnerability (Morais et al., 2021; Sampson et al., 2016). These distinctions should be interpreted as differences in mechanistic weighting rather than mutually exclusive biological systems.

This convergence–divergence framework provides the mechanistic foundation for evaluating dietary bioactives in the subsequent sections. Rather than asking whether a particular compound is inherently “anti-amyloid” or “anti-inflammatory,” the relevant question is how its interaction with the microbiome and microbial metabolites modifies the interconnected metabolic, immune, redox, and neuronal networks within the pathological context of AD or PD (Cryan et al., 2019; Morais et al., 2021).

## 7 Integrated mechanistic model of the gut–brain axis in neurodegeneration

The preceding evidence indicates that dietary bioactives can influence neurodegenerative processes through interconnected effects on gut ecology, microbial metabolism, systemic physiology, and CNS signaling (Cryan et al., 2019; Morais et al., 2021). Polyphenols and related phytochemicals may act directly on host pathways, indirectly through microbial biotransformation, or through both mechanisms simultaneously (Cardona et al., 2013; Ozdal et al., 2016). Nanoparticle-based formulations and synbiotic strategies may further modify these interactions by altering bioavailability, intestinal exposure, microbial ecology, and metabolite production (Westfall et al., 2017).

The resulting framework is best represented as a dynamic five-node network rather than a strictly linear pathway: dietary bioactive exposure → microbiome remodeling → microbial metabolite reprogramming → integrated systemic signaling → disease-specific neural outcomes. Bidirectional feedback between the brain and gut connects these nodes and allows disease progression itself to modify intestinal physiology (Figure 2; Morais et al., 2021; Quigley, 2017).

**FIGURE 2:**
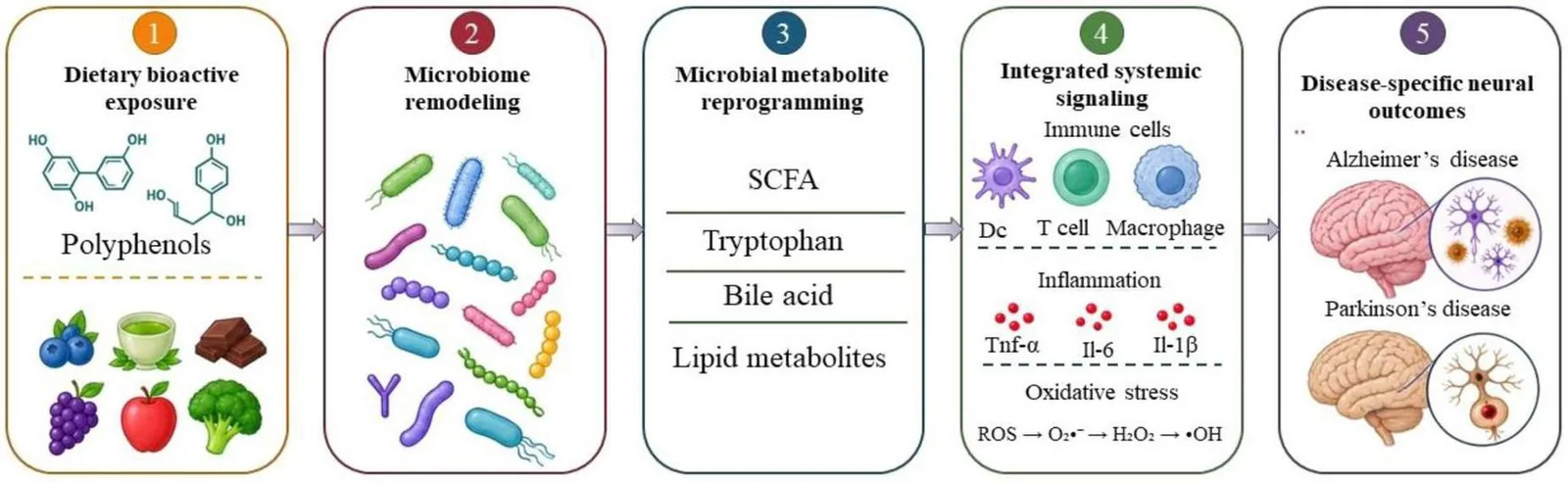
Integrated mechanistic model of the gut–brain axis in neurodegeneration. The model is organized as a nonlinear, five-node network. Node 1 (dietary bioactive exposure) captures the intake of polyphenols and related bioactives that serve as substrates for microbial metabolism. Node 2 (microbiome remodeling) reflects shifts in microbial community structure and diversity. Node 3 (microbial metabolite reprogramming) represents changes in SCFAs, tryptophan-derived metabolites, bile acids, and lipid- and energy-related metabolites, which act as the central metabolic–immune hub of the network. Node 4 (integrated systemic signaling) encompasses immune tone, inflammatory mediators, oxidative stress, and metabolic state that relay gut-derived signals toward the central nervous system (CNS). Node 5 (disease-specific neural outcomes) denotes the downstream neuroinflammatory and neurodegenerative consequences that are differentially organized in AD and PD. Bidirectional connections between the gut and brain indicate feedback through autonomic dysfunction, altered motility, diet, and systemic inflammation. This network emphasizes the microbiome as a biological amplifier that translates dietary and pharmacological inputs into immunometabolic signals shaping CNS pathology, and it highlights microbial metabolites as candidate biomarkers and precision-intervention targets.

### 7.1 Dietary bioactives as primary modulators of gut ecology

Dietary bioactives, including curcumin, resveratrol, quercetin, EGCG, salvianolic acids, anthocyanins, chicoric acid, and complex polyphenol mixtures, represent the initial intervention point within the framework (Cardona et al., 2013; Morais et al., 2021; Ozdal et al., 2016). Across AD and PD models, these compounds can alter microbial composition, promote beneficial commensals such as *Lactobacillus*, *Bifidobacterium*, *Blautia*, and *Allobaculum*, and suppress dysbiosis-associated populations, including members of Proteobacteria, Enterobacteriaceae, and Helicobacter (Cattaneo et al., 2017; Sampson et al., 2016).

The biological consequences are unlikely to depend on a single taxon. Instead, dietary bioactives appear to influence broader ecological properties, including microbial diversity, community stability, SCFA-producing capacity, and the balance between potentially protective and pro-inflammatory microbial functions (Cryan et al., 2019; Morais et al., 2021). These changes establish the microbial environment through which dietary compounds can exert downstream metabolic and immune effects.

Delivery systems may modify this first node. Curcumin- or resveratrol-based nanoparticles can improve compound stability, bioavailability, and intestinal exposure and may consequently enhance microbiome remodeling and downstream neuroprotective effects, as reported by Yang et al. (2023a) and Mu et al. (2026).

### 7.2 Microbial metabolite reprogramming as the central signaling hub

Microbiome remodeling constitutes the transition from ecological change to biochemical signaling. Altered microbial communities modify the production, transformation, and availability of metabolites that influence intestinal, systemic, and neural physiology (Cryan et al., 2019; Morais et al., 2021).

SCFAs, particularly acetate, propionate, and butyrate, contribute to epithelial barrier integrity, immune regulation, metabolic signaling, and microglial homeostasis (Dalile et al., 2019; Erny et al., 2015). Tryptophan-derived metabolites, including indole derivatives and kynurenine-pathway intermediates, participate in host–microbe communication and neuroimmune regulation (Agus et al., 2018; Rothhammer and Quintana, 2019). Lipid-associated, redox-active, and other microbial metabolites can additionally influence insulin sensitivity, mitochondrial function, oxidative stress, and neuronal energy metabolism (Cani et al., 2019; Morais et al., 2021).

These metabolites therefore form a metabolic–immune signaling hub rather than a collection of independent mediators. Their effects depend on concentration, tissue distribution, receptor engagement, host metabolic state, and disease context (Agus et al., 2018; Dalile et al., 2019). This node provides a mechanistic explanation for how relatively broad microbiome changes can generate specific systemic and CNS effects (Cryan et al., 2019; Morais et al., 2021).

### 7.3 Peripheral metabolic and immune reprogramming

Microbial metabolites subsequently influence peripheral physiology through interacting metabolic, immune-inflammatory, and redox pathways. These axes should not be considered mutually exclusive.

In AD models, metabolic regulation is particularly prominent. Changes in microbial signaling can influence insulin sensitivity, glucose metabolism, and lipid homeostasis, with downstream effects on AKT/GSK3β signaling and amyloid/tau-related processes (Lamichhane et al., 2024; Yang et al., 2023a). Such effects are especially evident in obesity-associated AD models, in which resveratrol- and curcumin-based interventions have been associated with improved metabolic balance and reduced Aβ pathology (Lamichhane et al., 2024; Yang et al., 2023a). At the same time, NF-κB-mediated inflammation and NRF2-dependent antioxidant responses remain integral to these effects.

In PD models, intestinal immune-inflammatory signaling is more prominently represented. Microbiome remodeling can attenuate TLR4/MyD88/NF-κB activation, reduce pro-inflammatory cytokines such as TNF-α, IL-6, and IL-1β, and improve intestinal barrier integrity, thereby limiting endotoxin translocation (Cai et al., 2023; Tao et al., 2023; Zhong et al., 2023). Modulation of inflammasome pathways, including AIM2/caspase-1 signaling, may further reduce inflammatory amplification (Tao et al., 2023; Zhong et al., 2022). These mechanisms are particularly relevant in toxin-induced models such as MPTP and rotenone, in which intestinal and systemic inflammatory disturbances contribute to dopaminergic neuronal injury.

### 7.4 Neuroimmune interface and disease-specific brain vulnerability

Peripheral metabolic, immune, and redox signals ultimately converge at the neuroimmune interface. This node encompasses blood–brain barrier regulation, microglial activation, neuroinflammation, oxidative stress, mitochondrial homeostasis, and neuronal signaling.

In AD, systemic metabolic disturbances and inflammatory mediators may compromise blood–brain barrier integrity and promote microglial activation, oxidative stress, Aβ accumulation, and tau hyperphosphorylation. AKT/GSK3β, NRF2, NF-κB, and BDNF-associated signaling can influence these processes and contribute to neuronal and synaptic resilience (Su et al., 2022; Westfall et al., 2019). The downstream vulnerability is concentrated particularly within cortical and hippocampal networks.

In PD, gut-derived inflammatory signals and microbial products can influence CNS immune signaling through systemic and neural communication routes, including vagal pathways. These signals interact with mitochondrial dysfunction, oxidative stress, α-synuclein pathology, and inflammatory signaling to increase the vulnerability of dopaminergic neurons in the substantia nigra pars compacta. SIRT1/NRF2-associated antioxidant mechanisms and suppression of TLR4/MyD88/NF-κB signaling represent important neuroprotective mechanisms in this context (Wang et al., 2022; Zhong et al., 2022). These findings indicate that similar upstream gut-derived signals are interpreted differently by distinct neural circuits, producing cortical–hippocampal degeneration in AD and nigrostriatal degeneration in PD.

Thus, a common set of peripheral signals can be translated into different CNS phenotypes because the vulnerable cellular populations and pathological substrates differ between AD and PD.

### 7.5 Neural outcomes and bidirectional feedback

The final node encompasses disease-specific neural and behavioral outcomes. In AD, modulation of the preceding nodes is primarily reflected in reduced Aβ/tau pathology, preservation of synaptic function, and improvements in learning and memory (Cattaneo et al., 2017; DeTure and Dickson, 2019). In PD, corresponding effects include preservation of nigrostriatal dopaminergic neurons, improvement in motor performance, and attenuation of gastrointestinal dysfunction (Morais et al., 2021; Sampson et al., 2016).

Importantly, this final node is not terminal in a biological sense. Neurodegeneration can itself modify intestinal physiology through autonomic dysfunction, altered gastrointestinal motility, enteric nervous system abnormalities, dietary changes, and systemic inflammatory responses (Morais et al., 2021; Quigley, 2017). These changes can further disturb the intestinal microbial ecosystem and reinforce dysbiosis. The gut–brain axis therefore operates as a bidirectional feedback system, in which microbiome-derived signals influence neurodegeneration while neurodegenerative processes reciprocally modify gut physiology (Cryan et al., 2019; Morais et al., 2021).

Experimental interventions such as FMT, synbiotic supplementation, and sustained polyphenol administration provide evidence that this feedback loop is potentially modifiable (Sampson et al., 2016; Westfall et al., 2017). For example, FMT from resveratrol-treated donors has reproduced anti-inflammatory and neuroprotective phenotypes in PD models, supporting a functional contribution of the microbial community to disease modulation (Tao et al., 2023; Westfall et al., 2019).

### 7.6 Unified model summary

Accordingly, the integrated model may be understood as five interconnected nodes rather than a linear progression (Cryan et al., 2019; Morais et al., 2021). The first node concerns dietary bioactive exposure, in which polyphenols and related phytochemicals act directly on host tissues and, indirectly, shape the intestinal microbiome (Cardona et al., 2013; Ozdal et al., 2016). The second node is microbiome remodeling, defined by alterations in microbial diversity, community structure, and functional capacity, including changes in populations linked to beneficial effects, SCFAs production, and dysbiosis (Cattaneo et al., 2017; Sampson et al., 2016). The third node is metabolite reprogramming, where SCFAs, tryptophan-derived metabolites, and lipid-, energy-, and redox-associated metabolites form central biochemical interfaces connecting microbial ecology to host physiology (Agus et al., 2018; Dalile et al., 2019; Morais et al., 2021). The fourth node comprises coordinated systemic signaling, involving interacting metabolic, immune-inflammatory, oxidative, and mitochondrial pathways across both gut and peripheral tissues, with their relative impacts varying between AD and PD (Cattaneo et al., 2017; Morais et al., 2021; Sampson et al., 2016). The fifth node involves disease-specific neural outcomes, where convergent peripheral signals interact with distinct molecular and neuroanatomical susceptibilities, thereby mainly driving Aβ/tau-related cognitive impairment in AD and α-synuclein-related dopaminergic degeneration and motor dysfunction in PD (DeTure and Dickson, 2019; Sampson et al., 2016). These five nodes are linked by reciprocal feedback rather than structured as an irreversible sequence (Cryan et al., 2019; Quigley, 2017). This arrangement helps account for how chemically diverse interventions can yield convergent neuroprotective effects even when their primary molecular targets differ (Cardona et al., 2013; Morais et al., 2021). Although many interventions ultimately act on a conserved network of microbial, metabolic, immune, redox, and mitochondrial signals, the resulting neurological phenotype still depends on disease-specific vulnerability (Morais et al., 2021).

### 7.7 Translational implications of the integrated model

The integrated framework has several implications for future therapeutic strategies. First, the gut microbiome may function as a biological amplifier and determinant of treatment responsiveness, rather than simply a passive biomarker of disease (Cryan et al., 2019; Morais et al., 2021). The biological effects of polyphenols can depend on microbial composition and metabolic capacity, suggesting that baseline microbiome characteristics may contribute to inter-individual variability in response (Cardona et al., 2013; Ozdal et al., 2016).

Second, microbial metabolites provide candidate mechanistic biomarkers and potential therapeutic targets. SCFAs, indole derivatives, kynurenine-pathway intermediates, bile acid derivatives, and other microbial metabolites may provide measurable indicators of microbiome functional activity and treatment response (Agus et al., 2018; Dalile et al., 2019; Morais et al., 2021). Their therapeutic relevance, however, requires validation through longitudinal and receptor-level studies rather than inference from associations alone.

Third, the model allows for disease-specific intervention strategies. In AD, interventions that improve metabolic function and insulin signaling while also lowering oxidative and inflammatory stress may be especially pertinent to amyloid and tau–related pathology (Cattaneo et al., 2017; DeTure and Dickson, 2019). In PD, approaches aimed at preserving intestinal barrier integrity, modulating gut-derived inflammatory signaling, addressing mitochondrial stress, and accounting for dopaminergic vulnerability may carry greater mechanistic significance (Morais et al., 2021; Sampson et al., 2016). These priorities are complementary rather than mutually exclusive.

Finally, formulation and combination strategies may increase the ability of dietary bioactives to affect the gut–brain axis. Nanoparticle delivery, synbiotics, probiotics, and other microbiome-targeted methods could potentially alter exposure, microbial metabolism, and host signaling at the same time (Westfall et al., 2017). Still, the translational value of these approaches hinges on demonstrating consistent microbiome–metabolite–host relationships in humans and on establishing whether preclinical neuroprotective effects can be sustained across clinically meaningful time frames.

Overall, the five-node model reframes the gut microbiome as an active regulatory interface linking nutrition, microbial metabolism, systemic physiology, and neurodegeneration (Cryan et al., 2019; Morais et al., 2021). It also provides a mechanistic basis for disease-tailored precision strategies in which the same upstream gut–brain architecture can be targeted while the intervention is aligned with the distinct molecular, metabolic, immune, and neuroanatomical vulnerabilities of AD and PD.

## 8 Discussion

The current synthesis of preclinical and translational evidence establishes the gut–brain axis as a convergent mechanistic framework by which dietary bioactives, particularly polyphenols, their derived metabolites, and nanoparticle-based delivery systems, modulate neurodegenerative processes in AD and PD (Cryan et al., 2019; Morais et al., 2021). Across a spectrum of experimental systems, including rodent models (APP/PS1, 5xFAD, MPTP, rotenone), transgenic organisms, and Drosophila paradigms, a consistent and biologically coherent pattern emerges: gut microbiota remodeling functions not merely as a secondary correlate of neurodegeneration but as a functionally upstream regulator of systemic immune tone, metabolic state, and ultimately, CNS pathology (Cattaneo et al., 2017; Sampson et al., 2016; Vogt et al., 2017).

An additional layer of complexity is provided by epigenetic regulation, which may connect microbiota-derived metabolites and dietary bioactives with persistent changes in host gene expression (Canani et al., 2011; Krautkramer et al., 2016). Microbial metabolites, particularly SCFAs and tryptophan-derived compounds, can influence chromatin accessibility, histone acetylation, DNA methylation, and non-coding RNA networks, thereby modifying inflammatory, metabolic, antioxidant, mitochondrial, and synaptic pathways (Agus et al., 2018; Krautkramer et al., 2016; Qin et al., 2022). Polyphenols may also directly regulate epigenetic enzymes and transcriptional programs, including histone deacetylases, DNA methyltransferases, and microRNAs (Shankar et al., 2016). Thus, the gut–brain axis should be viewed not only as a microbial–metabolic–immune network but also as an epigenetically responsive regulatory system in which environmental and nutritional signals can be translated into relatively persistent changes in cellular phenotype (Cryan et al., 2019; Morais et al., 2021).

This convergence across diverse models and interventions, ranging from curcumin, resveratrol, EGCG, salvianolic acids, anthocyanins, and chicoric acid to engineered nanoparticles and synbiotic systems, suggests that neuroprotection is less dependent on compound-specific pharmacology and more strongly associated with the capacity to restore microbial ecosystem stability and metabolite homeostasis (Cardona et al., 2013; Ozdal et al., 2016; Westfall et al., 2017). In this context, the gut microbiome acts as a “biological amplifier,” translating dietary or pharmacological inputs into systemic immunometabolic signals that shape neuroinflammatory and neurodegenerative trajectories (Cryan et al., 2019; Morais et al., 2021).

### 8.1 Microbiome modulation as a shared upstream determinant

A central observation across both AD and PD datasets is the recurrence of a conserved microbial signature following effective interventions (Cattaneo et al., 2017; Morais et al., 2021; Sampson et al., 2016). Beneficial taxa, such as Lactobacillus, Bifidobacterium, Blautia, Allobaculum, and *Ruminiclostridium*, are consistently enriched, whereas pro-inflammatory or dysbiosis-associated taxa, including Proteobacteria, Enterobacteriaceae, Helicobacter, Parabacteroides, and shifts associated with Muribaculaceae, are reduced (Cattaneo et al., 2017; Sampson et al., 2016).

This pattern indicates that neuroprotective efficacy is tightly linked to the restoration of microbial ecological balance rather than to any single taxonomic alteration (Cryan et al., 2019; Morais et al., 2021). Instead of acting through highly specific microbial targets, most interventions appear to normalize broader ecological properties, such as diversity, the Firmicutes/Bacteroidota ratio, and SCFA-producing capacity (Cattaneo et al., 2017; Sampson et al., 2016). This reinforces the concept of a “conserved functional dysbiosis features” shared across neurodegenerative states (Cryan et al., 2019; Morais et al., 2021).

Importantly, mechanistic causality is supported by microbiota manipulation experiments. Westfall et al. (2019) demonstrated in a Drosophila AD model that synbiotic supplementation improved survival, reduced amyloid burden, and ameliorated metabolic stress parameters via gut–brain axis signaling pathways, including PPARγ-associated regulation. Similarly, Xu et al. (2020) showed in a PD Drosophila model that EGCG-mediated neuroprotection is abolished when gut microbiota are disrupted by antibiotics and partially restored through bacterial supplementation. These findings provide strong evidence that the microbiome is not merely a biomarker but a causal mediator of neurobehavioral outcomes.

### 8.2 Metabolite-mediated signaling as the functional bridge

Microbial metabolites provide a crucial mechanistic link connecting remodeling of the gut ecosystem with systemic and central nervous system outcomes. Across the studies reviewed, three main metabolite groups are consistently implicated in gut–brain communication: short-chain fatty acids, tryptophan-derived metabolites, and energy- and lipid-related metabolites (Agus et al., 2018; Cryan et al., 2019; Dalile et al., 2019). Short-chain fatty acids, including acetate, propionate, and butyrate, are associated with maintenance of intestinal barrier integrity, immune regulation, metabolic signaling, and regulation of microglial activity (Agus et al., 2018; Dalile et al., 2019). Tryptophan-derived metabolites, including indole derivatives and intermediates from the kynurenine pathway, contribute to host–microbe communication and neuroimmune regulation through mechanisms that involve receptors such as the aryl hydrocarbon receptor (Agus et al., 2018; Rothhammer and Quintana, 2019). Energy- and lipid-related metabolites, particularly relevant to metabolically disturbed AD models, can affect neuronal energy homeostasis, insulin signaling, lipid balance, and synaptic function (Agus et al., 2018; Cani et al., 2019).

These metabolites also establish a mechanistic connection to epigenetic control. Butyrate and related SCFAs can suppress class I and II histone deacetylases, leading to higher levels of histone acetylation and modifying the transcription of genes that participate in inflammation, oxidative stress, metabolism, and neuronal plasticity (Cryan et al., 2019; Krautkramer et al., 2016). Microbiota-driven shifts in one-carbon metabolism can modulate the supply of methyl donors and thereby influence DNA and histone methylation (Krautkramer et al., 2016). Metabolites derived from tryptophan may govern transcription via aryl hydrocarbon receptor-dependent pathways and may engage cellular mechanisms that shape chromatin organization (Rothhammer and Quintana, 2019). Accordingly, microbial metabolites can act as epigenetic intermediates through which alterations in intestinal ecology are converted into sustained changes in host cellular responses (Krautkramer et al., 2016).

Dietary polyphenols may strengthen these effects by directly altering epigenetic enzymes and non-coding RNA networks. Curcumin, resveratrol, quercetin, and EGCG have been studied as modulators of DNA methyltransferases, histone-modifying enzymes, sirtuins, and microRNAs within neurodegenerative and inflammatory settings (Li et al., 2026; Shankar et al., 2016). These actions may align with NRF2, NF-κB, SIRT1, Akt, and mitochondrial signaling, resulting in an integrated regulatory network rather than a separate epigenetic pathway (Li et al., 2026) .

In AD models, SCFAs and lipid-related metabolites converge on insulin signaling dysregulation and amyloid/tau pathology through key molecular pathways, including AKT/GSK3β signaling, NRF2-mediated antioxidant responses, and NF-κB-driven inflammatory cascades (Yang et al., 2023a). Collectively, these pathways regulate amyloid-β production, tau phosphorylation, oxidative stress levels, and synaptic integrity, positioning microbial metabolites as upstream modulators of core AD pathology. In contrast, PD models demonstrate a stronger dependence on immune-modulatory SCFA effects and tryptophan-associated inflammatory regulation, with key signaling mechanisms involving TLR4/MyD88/NF-κB activation, SIRT1/NRF2 antioxidant pathways, and suppression of inflammasome activity, particularly the AIM2/caspase-1 axis (Tao et al., 2023; Zhong et al., 2022). These findings collectively indicate that microbial metabolites do not exert uniform effects across neurodegenerative diseases; rather, they are selectively integrated into disease-specific molecular networks that determine whether metabolic, inflammatory, or neurodegenerative pathways dominate the downstream CNS response.

### 8.3 Epigenetic regulation as a convergence layer between the gut and brain

Epigenetic mechanisms offer a credible molecular link between environmental exposure, microbial metabolism, and enduring vulnerability to neurodegeneration (Krautkramer et al., 2016; Zhang et al., 2023). In contrast to genetic mutations, epigenetic modifications can change in a responsive manner in relation to diet, inflammation, oxidative stress, microbial metabolites, and the cell’s metabolic condition (Coppedè, 2021; Krautkramer et al., 2016). As a result, they are especially pertinent to gut–brain axis biology, where the microbial and nutritional inputs are continuously shifting and can reshape host transcriptional programs (Altan and Ihlamur, 2026; Zhang et al., 2023).

Three principal epigenetic mechanisms are of particular relevance. First, histone acetylation and deacetylation can be modulated by microbial metabolites, with a special emphasis on SCFAs, as well as by host sirtuin activity (Coppedè, 2021; Krautkramer et al., 2016). Greater histone acetylation may shift the expression of inflammatory and neuroprotective genes, while SIRT1-dependent deacetylation can govern oxidative-stress responses, mitochondrial performance, and inflammatory signaling (Coppedè, 2021; Zhang et al., 2023). Second, DNA methylation can be shaped by the metabolic availability of methyl donors and by alterations in one-carbon metabolism, with potential effects on the long-term expression of genes that support neuronal function, inflammation, and metabolic regulation (Coppedè, 2021; Jiang et al., 2016). Third, non-coding RNAs, especially microRNAs, can integrate metabolic and inflammatory cues and modulate the post-transcriptional expression of genes that contribute to synaptic plasticity, mitochondrial homeostasis, neuroinflammation, and neuronal survival (Zhang et al., 2023).

In AD, these processes may affect the expression of genes involved in Aβ processing, tau phosphorylation, insulin signaling, antioxidant defenses, and synaptic plasticity (Jiang et al., 2016; Zhang et al., 2023). In PD, epigenetic regulation may influence genes and pathways related to α-synuclein homeostasis, mitochondrial activity, oxidative stress, inflammatory signaling, and the survival of dopaminergic neurons (Zhang et al., 2023). Notably, these disease-specific effects take place within a common epigenetic framework. Consequently, epigenetic regulation may help account for how related microbial or metabolic signals can produce distinct neurological phenotypes in AD and PD (Zhang et al., 2023).

This relationship may also be bidirectional. Inflammation associated with neurodegeneration, dysregulated mitochondrial metabolism, oxidative stress, and shifts in dietary behavior can, in turn, alter epigenetic states (Coppedè, 2021; Zhang et al., 2023). These alterations may subsequently influence intestinal barrier function, immune activity, and microbial ecology, creating a feedback loop between microbiota, host epigenetics, and CNS pathology (Altan and Ihlamur, 2026; Nohesara et al., 2024). This concept extends the gut–brain axis from a communication pathway to a microbiome–metabolite–epigenome–brain regulatory network.

### 8.4 Divergence of downstream brain pathways in AD and PD

Despite substantial overlap in upstream microbiome and metabolite changes, downstream central nervous system responses in AD and PD reflect both shared regulatory mechanisms and distinct disease-specific vulnerability architectures. In AD, the pathological cascade is defined by extracellular amyloid-β accumulation, intracellular tau hyperphosphorylation, progressive synaptic impairment, and cognitive decline (Bloom, 2014; DeTure and Dickson, 2019). These events involve AKT/GSK3β signaling, which modulates amyloid processing and tau phosphorylation, alongside NRF2-mediated antioxidant defense, NF-κB-driven neuroinflammatory signaling, and BDNF-associated pathways that promote synaptic plasticity and neuronal resilience (Bloom, 2014; Coppedè, 2021). However, these pathways are not exclusive to AD. NRF2 and NF-κB, in particular, are broadly conserved regulators of oxidative stress and innate immune responses that are also implicated in PD, whereas Akt-dependent survival signaling can affect neuronal resilience across both disorders (Smajić et al., 2022; Zhang et al., 2023). PD is primarily marked by progressive degeneration of dopaminergic neurons in the substantia nigra pars compacta, α-synuclein-associated pathology, and motor dysfunction, often accompanied by gastrointestinal disturbances (Rocha et al., 2018; Sampson et al., 2016).

Its downstream signaling network includes SIRT1/NRF2-mediated control of oxidative stress and mitochondrial homeostasis, TLR4/MyD88/NF-κB-mediated inflammatory signaling, inflammasome-linked processes such as AIM2/caspase-1-mediated pyroptosis, and Akt-dependent neuronal survival pathways (Sampson et al., 2016; Zhang et al., 2023). Several of these mechanisms substantially overlap with those implicated in AD. For instance, chronic NF-κB activation, defective NRF2-dependent antioxidant defenses, mitochondrial dysfunction, inflammatory signaling, and altered Akt activity can all drive neuronal injury in both conditions (Coppedè, 2021; Jiang et al., 2016). Similarly, microglial activation and peripheral immune signaling create shared neuroimmune interfaces through which gut-derived metabolic and inflammatory cues may modulate central nervous system vulnerability (Erny et al., 2015; Nohesara et al., 2024).

Accordingly, the key difference lies less in whether particular pathways are present or absent than in how they are weighted, their molecular context, and their downstream targets. In AD, interactions among metabolic dysfunction, insulin signaling, AKT/GSK3β activity, oxidative stress, and neuroinflammation are tightly linked to amyloid-β and tau pathology, as well as to the vulnerability of cortical and hippocampal networks (Arnold et al., 2018; Bloom, 2014; DeTure and Dickson, 2019).

In PD, inflammatory, mitochondrial, and redox disturbances are more tightly connected with alpha-synuclein pathology and with the selective susceptibility of nigrostriatal dopaminergic neurons (Sampson et al., 2016; Zhang et al., 2023). Accordingly, the same upstream signals, such as short-chain fatty acids, inflammatory mediators, oxidative stress, and altered microbial metabolites, may engage partly overlapping signaling networks, yet yield distinct pathological outcomes depending on the cellular and anatomical context (Agus et al., 2018; Dalile et al., 2019; Nohesara et al., 2024).

This convergence–divergence framework enables a more refined interpretation of the gut–brain axis in neurodegeneration. Rather than endorsing a rigid metabolic–amyloid model for AD and an immune–inflammatory model for PD, the current body of evidence suggests a common metabolic–immune–redox signaling framework that is differentially arranged in each condition (Coppedè, 2021; Nohesara et al., 2024). As a result, AD and PD share several potentially actionable molecular targets, including NRF2, NF-κB, Akt signaling, mitochondrial homeostasis, and microglial regulation, while differing in the pathological substrates and neuronal populations ultimately implicated (Jiang et al., 2016; Zhang et al., 2023). The gut–brain axis may therefore function as a common upstream regulatory system whose downstream effects are conditioned by disease-specific molecular and neuroanatomical vulnerabilities.

### 8.5 Role of formulation strategies: nanoparticles and synbiotics

A major translational advancement emerging from the reviewed literature is the enhanced efficacy of engineered delivery systems, particularly nanoparticle-based formulations and synbiotic interventions, which substantially improve the therapeutic potential of polyphenols in neurodegenerative disease models (Anand et al., 2007; Li et al., 2021; Prasad et al., 2014). Nanoparticles, such as curcumin-loaded chitosan/tripolyphosphate (CS/TPP) systems and brain-targeted resveratrol carriers, offer several key advantages, including increased oral bioavailability, improved metabolic stability, enhanced permeability across the blood-brain barrier (BBB), and more sustained and consistent modulation of gut microbial composition (Anand et al., 2007; Li et al., 2021; Prasad et al., 2014). These properties enable a stronger and more durable impact on downstream signaling pathways. For instance, Yang et al. (2023a) demonstrated that resveratrol-loaded, brain-targeted nanoparticles significantly ameliorate insulin resistance, reduce amyloid-β and tau pathology, and reshape the gut microbiota toward a neuroprotective profile. Similarly, Mu et al. (2026) reported that curcumin nanoparticles more effectively activate Akt signaling, inhibit neuronal apoptosis, and induce more pronounced microbiome restructuring compared to free curcumin, highlighting the importance of delivery efficiency in modulating the gut–brain axis.

Synbiotic strategies further expand this therapeutic paradigm by combining probiotics with polyphenol-rich substrates to achieve synergistic effects on microbial ecology and host physiology. By simultaneously supplying beneficial microbial strains and bioactive compounds that support their growth and metabolic activity, synbiotics enhance both microbiome stability and metabolite production. Westfall et al. (2019) demonstrated that such a synbiotic formulation improves survival, reduces amyloid burden, and stabilizes metabolic and oxidative stress pathways in AD models through coordinated gut–brain axis signaling. Collectively, these findings support a shift toward systems-level therapeutic approaches that integrate microbiome modulation, metabolic regulation, and targeted delivery, rather than relying on single-compound or single-pathway interventions.

### 8.6 Translational implications and therapeutic positioning

The available evidence supports a paradigm shift from a traditionally CNS-centric view of neurodegeneration toward a microbiome-centered and metabolically integrated framework. Within this model, three major translational implications emerge. First, microbiome responsiveness may serve as a predictive biomarker of therapeutic efficacy, as baseline gut microbial composition appears to influence individual responses to polyphenols and other microbiota-targeted interventions. This underscores the importance of stratified or precision medicine approaches that account for inter-individual variability in gut ecology. Second, metabolite-based biomarkers, particularly SCFAs, indole derivatives, and kynurenine pathway intermediates, offer a quantifiable and mechanistically relevant link between dietary or pharmacological interventions and neurobiological outcomes. These metabolites may function not only as diagnostic indicators but also as early markers of treatment response and disease modulation. Third, there is a strong rationale for combination therapies, as multimodal strategies integrating polyphenols with antibodies, nanoparticle delivery systems, probiotics, or metabolic regulators consistently demonstrate superior efficacy to single-agent approaches. This likely reflects their capacity to simultaneously modulate microbial composition, systemic inflammatory processes, and central signaling pathways.

### 8.7 Limitations of current evidence

Despite the strong and consistent mechanistic convergence observed across studies, several limitations warrant consideration. First, the field remains dominated by preclinical research, including rodent models (e.g., APP/PS1, 5xFAD, MPTP, rotenone) and invertebrate systems such as Drosophila. Although these models provide valuable mechanistic insight into gut–brain interactions, they do not fully recapitulate the complexity, heterogeneity, and environmental variability of human neurodegenerative disease. Differences in diet, microbiome composition, immune function, and lifespan between model organisms and humans limit direct clinical extrapolation.

In addition, substantial methodological heterogeneity persists across studies. Variability in microbiome sequencing methods (e.g., 16S rRNA versus shotgun metagenomics), taxonomic resolution, and analytical pipelines introduces inconsistencies that hinder robust cross-study comparison. Differences in animal strain, disease induction method, intervention dose, and treatment duration further complicate synthesis and standardization. These issues are compounded by inconsistent reporting, as some studies provide detailed microbial and pathway-level data, while others report only broad compositional shifts.

Another limitation is incomplete and uneven metabolomic profiling. Although SCFAs are frequently measured and consistently implicated, other metabolite classes, such as tryptophan-derived compounds, bile acids, and lipid mediators, are less systematically evaluated. This fragmented approach limits a comprehensive understanding of the metabolic networks underlying gut–brain communication. Furthermore, causal links between specific metabolites and receptor-level signaling in the brain remain insufficiently validated in many studies.

Finally, most studies rely on relatively short intervention periods and lack long-term follow-up. Consequently, it remains unclear whether microbiome remodeling and associated neuroprotective effects are durable or require continuous intervention. The absence of longitudinal data also limits understanding of disease progression and the relative value of early versus late intervention.

### 8.8 Future directions

Future research should prioritize integrated multi-omics approaches that simultaneously characterize the microbiome, metabolome, epigenome, transcriptome, and CNS phenotype. Longitudinal human studies should determine whether microbiota-derived metabolites precede changes in DNA methylation, histone modifications, chromatin accessibility, or microRNA expression and whether these molecular changes predict cognitive or motor outcomes.

Mechanistic studies should specifically examine whether microbial metabolites such as butyrate and indole derivatives directly modify epigenetic enzymes or transcriptional programs relevant to AD and PD. Experiments combining microbiota depletion or transplantation with metabolite supplementation, epigenetic profiling, receptor-specific manipulation, and genetic validation would provide stronger evidence for causality.

Future studies should also determine whether polyphenols exert disease-specific epigenetic effects and whether these effects depend on microbial biotransformation. Particular attention should be given to SIRT1, histone deacetylases, DNA methyltransferases, NRF2, NF-κB, and disease-relevant microRNAs because these regulatory nodes intersect metabolic, inflammatory, oxidative, and neuronal pathways.

Finally, precision strategies should integrate baseline microbiome composition, microbial metabolic capacity, epigenetic state, diet, physical activity, medication exposure, and disease stage. Such an approach could move the field toward a microbiome–metabolite–epigenome–brain precision framework, in which dietary bioactives and microbiota-directed interventions are selected according to both microbial functionality and host molecular vulnerability.

### 8.9 Conclusion

The available preclinical evidence supports the gut–brain axis as a dynamic regulatory interface linking dietary bioactives, microbial ecology, microbial metabolites, systemic metabolism, immune signaling, epigenetic regulation, and neurodegenerative pathology in AD and PD. Although AD and PD are characterized by distinct pathological substrates, namely Aβ/tau pathology and cognitive network vulnerability in AD and α-synuclein accumulation with selective dopaminergic degeneration in PD, they share substantial upstream mechanisms involving gut dysbiosis, impaired microbial metabolite signaling, intestinal barrier dysfunction, oxidative stress, mitochondrial impairment, and chronic neuroinflammation.

Dietary polyphenols, including curcumin, resveratrol, quercetin, EGCG, anthocyanins, salvianolic acids, and chicoric acid, appear capable of influencing this interconnected network through both direct host actions and microbiota-dependent mechanisms. Restoration of microbial ecological balance and SCFA production may reduce systemic inflammatory tone, improve metabolic homeostasis, reinforce intestinal barrier integrity, and modulate CNS immune and neuronal responses. Microbial biotransformation further determines the bioavailability and biological activity of several phytochemicals, positioning the microbiome as an active determinant of dietary bioactive efficacy rather than merely a passive target.

Epigenetic regulation provides an additional mechanistic layer within this framework. Microbial metabolites, particularly SCFAs and tryptophan-derived compounds, can influence histone acetylation, DNA methylation, chromatin accessibility, and non-coding RNA networks, whereas polyphenols may directly modulate epigenetic enzymes and transcriptional regulators. These mechanisms offer a plausible explanation for how transient nutritional and microbial signals can produce sustained changes in inflammatory, metabolic, antioxidant, mitochondrial, and neuronal pathways. Thus, the gut–brain axis can be conceptualized as a microbiome–metabolite–epigenome–brain network, rather than simply a pathway of microbial communication with the CNS.

The comparative evidence further indicates that disease specificity emerges primarily at the level of downstream vulnerability rather than through completely distinct upstream mechanisms. In AD, gut-derived metabolic and epigenetic signals may interact particularly strongly with insulin resistance, AKT/GSK3β signaling, Aβ/tau pathology, and cortical–hippocampal dysfunction. In PD, inflammatory, mitochondrial, redox, and epigenetic signals may interact more strongly with α-synuclein pathology and the selective vulnerability of nigrostriatal dopaminergic neurons. These distinctions support disease-tailored interventions while preserving the concept of a shared gut–brain regulatory architecture.

Nanoparticle delivery, probiotics, synbiotics, and other microbiota-targeted strategies may enhance the therapeutic potential of dietary bioactives by modifying bioavailability, microbial exposure, metabolite production, and host signaling simultaneously. Nevertheless, current evidence remains predominantly preclinical, and substantial heterogeneity in experimental models, microbiome methodologies, metabolomic characterization, epigenetic assessment, dosing, and intervention duration limits direct clinical translation. In particular, causal relationships among specific microbial taxa, metabolites, epigenetic modifications, and neurological outcomes remain insufficiently established.
